# Recent Advances in Perioperative Immunotherapies in Lung Cancer

**DOI:** 10.3390/biom13091377

**Published:** 2023-09-12

**Authors:** Shota Fukuda, Kenichi Suda, Akira Hamada, Yasuhiro Tsutani

**Affiliations:** Division of Thoracic Surgery, Department of Surgery, Kindai University Faculty of Medicine, 377-2 Ohno-Higashi, Osakasayama 589-8511, Japan; shota-fukuda@med.kindai.ac.jp (S.F.); a-hamada@med.kindai.ac.jp (A.H.); tsutani@med.kindai.ac.jp (Y.T.)

**Keywords:** non-small cell lung cancer (NSCLC), neoadjuvant treatment, adjuvant treatment, stage migration, ICI combination therapies, biomarker, programmed cell death ligand 1 (PD-L1)

## Abstract

Several clinical trials have been revolutionizing the perioperative treatment of early-stage non-small cell lung cancer (NSCLC). Many of these clinical trials involve cancer immunotherapies with antibody drugs that block the inhibitory immune checkpoints programmed death 1 (PD-1) and its ligand PD-L1. While these new treatments are expected to improve the treatment outcome of NSCLC patients after pulmonary resection, several major clinical questions remain, including the appropriate timing of immunotherapy (neoadjuvant, adjuvant, or both) and the identification of patients who should be treated with neoadjuvant and/or adjuvant immunotherapies, because some early-stage NSCLC patients are cured by surgical resection alone. In addition, immunotherapy may induce immune-related adverse events that will require permanent treatment in some patients. Based on this fact as well, it is desirable to select appropriate patients for neoadjuvant/adjuvant immunotherapies. So far, data from several important trials have been published, with findings demonstrating the efficacy of adjuvant atezolizumab (IMpower010 trial), neoadjuvant nivolumab plus platinum-doublet chemotherapy (CheckMate816 trial), and several perioperative (neoadjuvant plus adjuvant) immunotherapies (AEGEAN, KEYNOTE-671, NADIM II, and Neotorch trials). In addition to these key trials, numerous clinical trials have reported a wealth of data, although most of the above clinical questions have not been completely answered yet. Because there are so many ongoing clinical trials in this field, a comprehensive understanding of the results and/or contents of these trials is necessary to explore answers to the clinical questions above as well as to plan a new clinical trial. In this review, we comprehensively summarize the recent data obtained from clinical trials addressing such questions.

## 1. Introduction

The clinical application of immune checkpoint inhibitors (ICIs) has revolutionized the treatment of advanced-stage non-small cell lung cancer (NSCLC) and small cell lung cancer. Several antibodies blocking the inhibitory immune checkpoints programmed death 1 (PD-1) and its ligand PD-L1 and other checkpoints such as cytotoxic T-lymphocyte associated antigen-4 (CTLA-4) have been developed. Anti-PD-1/PD-L1 inhibitor monotherapies and combination therapies with cytotoxic chemotherapy (with/without anti-angiogenic agents) and/or anti-CTLA-4 inhibitors are now standard front-line therapies for advanced-stage NSCLC without a targetable driver mutation [1]. One of the important features of ICI-based treatments is the so-called “long tail effect”, which contributes to the curative effects in some advanced-stage NSCLC patients.

After the exciting success of immunotherapies in the advanced-stage setting, the subsequent logical step was the clinical application of ICIs for patients with non-metastatic NSCLC to improve cure rates and prolong overall survival (OS). The PACIFIC trial demonstrated a significant improvement in progression-free survival (PFS) and OS in patients with unresectable clinical stage III NSCLC treated with durvalumab after concurrent chemoradiotherapy, with improvements even in 5-year OS [2]. Four phase III trials (IMpower010 (NCT02486718) [3], PEARLS (NCT02504372) [4], BR31 (NCT02273375), and ANVIL (NCT02595944)) are now evaluating the efficacy and the safety of atezolizumab, pembrolizumab, durvalumab, and nivolumab, respectively, after curative resection for NSCLCs.

In the neoadjuvant setting, several small-scale single-arm studies have reported good treatment efficacies of ICI monotherapies in early-stage NSCLC patients [5]. These results led to the initiation of clinical trials of neoadjuvant ICI monotherapies or combination therapies in early-stage NSCLC. Furthermore, several clinical trials with a perioperative setting (neoadjuvant plus adjuvant ICIs, the so-called sandwich regimens) are also ongoing. Thus, a great number of clinical trials are now underway in this field, and the results of these trials are reported at every major conference. In order to provide the best treatment for the patient in front of us in our daily clinical practice, and in order to build new evidence in the future, it is necessary to have a comprehensive understanding of the results of these trials. Therefore, in this review, we summarize the recent advances in adjuvant, neoadjuvant, and perioperative treatments using ICIs in patients with resectable NSCLC. To collect as much information as possible about the literature and clinical trials on neoadjuvant, adjuvant, and perioperative treatments for NSCLC, we searched PubMed using a combination of the following keywords: “lung cancer”, “NSCLC”, “adjuvant”, “neoadjuvant”, “perioperative treatment”, and “immune checkpoint inhibitors”. Of the articles found using these search terms, all available review articles were checked, and individual clinical trial information was evaluated using its original data and included in the reference list of this paper if they were determined to be pertinent.

Although many clinical trials have been conducted in this field, there is still insufficient biomarker analysis data that are useful for better patient selection. Although some studies reported the results of biomarker analysis such as tumor mutation burden, gene expression data, liquid biopsy, tumor-infiltrating immune cells, and gut microbiota, their usefulness has not yet been fully verified, therefore in this review, we focused on summarizing clinical trial data but not including biomarker studies.

## 2. Treatment Outcomes of Early-Stage NSCLC before the ICI Era

Radical resection together with lymph node dissection is the standard of care for most NSCLC patients with clinical stage I/II disease and some patients with clinical stage III disease. The clinical stages are mainly determined by enhanced computed tomography (CT) and FDG-PET/CT examination.

**Figure 1:**
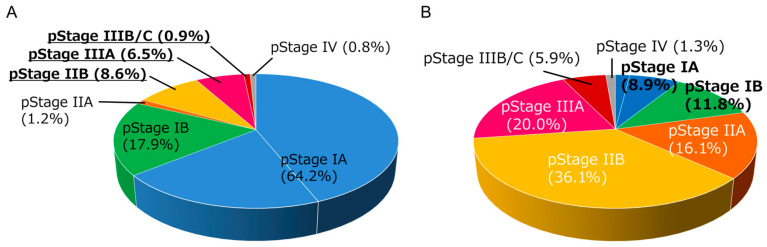
Distribution of pathological stages in non-small cell lung cancer (NSCLC) patients (n = 1590) who received lobectomy plus mediastinal lymph node dissection in our institution between 2007–2021. (**A**) Distribution of pathological stages in patients with clinical stage I NSCLC (n = 1285). Note that approximately 16% of patients experienced up-staging at pathological examination. (**B**) Distribution of pathological stages in patients with clinical stage II NSCLC (n = 305). Note that approximately 20% of patients had experienced down-staging into stage I at pathological examination.

After pulmonary resection, a detailed pathological examination enables a more accurate diagnosis of the spread of tumor cells (pathological stage, pStage). In NSCLC, there is a substantial discrepancy between clinical stage and pathological stage as exemplified in Figure 1A,B. Adjuvant treatment is applied in patients with good performance status in some pStage I patients and in pStage II–III patients. While a recent large-scale registry database analysis reported that approximately 60% of patients with pStage II or III disease received adjuvant treatment in the real-world setting in Japan [6], the primary analysis of this registry database showed that approximately 50% of pStage II patients and 75% of pStage III patients experienced disease recurrence or death within 5 years [7]. Therefore, the clinical application of new drugs in the neoadjuvant and/or adjuvant setting including tyrosine kinase inhibitors such as osimertinib [8] and ICIs, as summarized in this review, has been a research focus.

## 3. Overview of ICIs

Cytotoxic CD8-positive T cells are the main player in the anti-tumor immune response [9,10]. These T cells are activated only when the T cell receptor (TCR) recognizes the antigen/MHC-1 complexes on the surface of antigen-presenting cells (APC) (first signal) and the CD28 molecule on T cells binds to the B7 molecules (CD80/CD86) on the APC (second signal) at the same time. The CTLA-4 molecule, expressed on T cells, functions as a negative regulator of this T cell activation; CTLA-4 also binds to the B7 molecules on the APC at a higher affinity compared with CD28. Therefore, blockade of the CTLA-4 pathway, for example with anti-CTLA4 antibodies, promotes cytotoxic T cell activation [11].

Activated cytotoxic T cells infiltrate into the tumor area and kill tumor cells by enhancing pore formation in the tumor cell membrane and causing subsequent secretion of death-inducing granules containing granzymes, perforin, cathepsin C, and granulysin. Cytotoxic T cells also promote tumor cell death through Fas-FASL (Fas ligand) interactions. Tumor cell killing only takes a few minutes, and a single cytotoxic T cell can carry out serial or simultaneous killing of multiple tumor cells [12]. Immune cells, including cytotoxic T cells, have self-inhibitory mechanisms involving immune-checkpoint molecules that ensure the appropriate regulation of the immune response. PD-1 is one of the most important immune-checkpoint molecules that is expressed in exhausted cytotoxic T cells. Tumor cells exploit this inhibitory pathway by expressing the PD-1 ligands, PD-L1 or PD-L2, to induce an immunosuppressive state that facilitates tumor cell growth.

**Figure 2:**
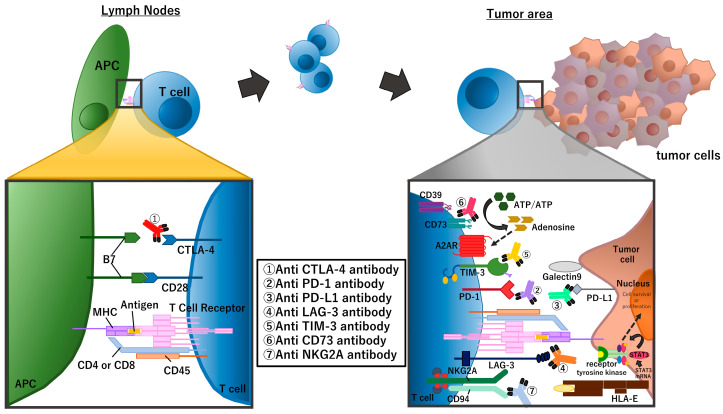
Summary of immune checkpoint molecules and the inhibitors targeting these molecules. The figure focuses on molecules that are targeted in clinical trials of perioperative treatment for NSCLC.

Many recent clinical studies of NSCLC in perioperative settings use one of the PD-1 inhibitors (nivolumab, pembrolizumab, cemiplimab, sintilimab, and toripalimab) or PD-L1 inhibitors (atezolizumab, durvalumab, and avelumab). In addition to PD-1/PD-L1, there are many other checkpoint molecules including the aforementioned CTLA-4, lymphocyte-activation gene 3 (LAG-3) [13], T-cell immunoglobulin and mucin domain-3 (TIM-3), and T-cell immunoreceptor with Ig and ITIM domains (TIGIT) and immune suppressive mechanisms including loss of stimulator of interferon genes (STING) expression [14] and the adenosine pathway (Figure 2).

## 4. Adjuvant ICI Treatments vs. Neoadjuvant ICI Treatments

In studies examining the efficacy of chemotherapy, both neoadjuvant [15] and adjuvant approaches [16,17] have shown superior efficacy in OS compared with surgery alone (but only by approximately 5%). However, because evidence for adjuvant chemotherapies was established earlier, several neoadjuvant trials using cytotoxic chemotherapies were terminated before maturation. Therefore, adjuvant chemotherapies have been considered the standard of care until the establishment of recent novel adjuvant/neoadjuvant treatments. However, in the new era of ICI treatments, the best strategy for surgical candidates with NSCLC, such as neoadjuvant immunotherapy vs. adjuvant immunotherapy vs. perioperative (neoadjuvant plus adjuvant, the so-called sandwich regimen) immunotherapy, remains unclear.

In an in vivo study of mice with tumors derived from highly metastatic breast cancer cell lines, mice treated with neoadjuvant immunotherapy and surgical resection had longer survival than those treated with upfront surgery followed by adjuvant immunotherapy [18]. While this in vivo observation was supported by the systemic expansion of tumor-specific cytotoxic T cells in peripheral blood and organs after neoadjuvant immunotherapy, whether this phenomenon also occurs in NSCLC patients is unclear. In the clinical setting of patients with resectable stage III or IV melanoma, event-free survival (EFS) was significantly longer for patients who received neoadjuvant pembrolizumab plus adjuvant pembrolizumab than patients who received adjuvant pembrolizumab alone [19]. However, whether this observation is applicable to NSCLC is unclear because the shapes of the EFS curves were quite different from the usual EFS curves after pulmonary resection. Here we summarize the possible advantages and disadvantages of neoadjuvant and adjuvant immunotherapies.

### 4.1. Advantages and Disadvantages of Neo-Adjuvant Immunotherapies

Theoretically, neoadjuvant immunotherapy will be able to prime a more effective immune reaction compared with adjuvant immunotherapy because of the abundant neoantigen and maintained lymphatic system around the primary tumor. Neoadjuvant immunotherapy also has advantages in terms of offering the earliest treatment against potential micrometastases, and treatments can be provided in patients with good compliance. Additionally, effective neoadjuvant immunotherapy will increase the resectability of the tumor (chance for R0 resection and/or avoidance of pneumonectomy). Furthermore, it is also possible that neoadjuvant treatment will provide some time for patients for tobacco cessation and respiratory rehabilitation. Neoadjuvant immunotherapies may also provide useful tumor samples for investigating drug tolerance mechanisms that have been widely studied for molecular-targeted agents [20,21] but not for immunotherapies.

There are also some disadvantages of neoadjuvant immunotherapy including delayed or missed surgical resection because of disease progression or severe adverse events including immune-related adverse events (irAEs). Neoadjuvant chemotherapy plus immunotherapy resulted in patients who could not receive surgical resection because of various reasons in phase III trials, comprising 15–20% of the overall enrolled patients [22,23,24]. There are also possibilities of severe irAEs after pulmonary resection that should be differentiated from surgical complications. As discussed above, some clinical N1–2 patients at preoperative image examination may have false-positive nodal status. Therefore, neoadjuvant immunotherapy may be an over-treatment for these patients.

### 4.2. Advantages and Disadvantages of Adjuvant Immunotherapies

As shown in Figure 1, there are substantial discrepancies between the clinical stage and the pathological stage in NSCLC patients. Therefore, adjuvant ICI treatment may have an advantage in that the treatment strategies can be determined on the basis of the most “correct” TNM staging. In the near future, it may be possible to identify the subgroup of patients who will be cured by surgery alone, for example, by circulating tumor DNA (ctDNA) detection [25,26,27]. Such information will lead to the administration of systemic treatments only for patients with a higher risk of recurrence in adjuvant ICI treatment strategies. Additionally, treatment strategies starting from pulmonary resection will ensure patients do not lose the opportunity to undergo surgical resection, the most robust treatment modality for local control of the primary tumor.

Notably, some patients may not be able to receive adjuvant treatment if the performance status is worsened after pulmonary resection partially from post-surgical complications. Studies showed that only 66% of patients received pre-planned adjuvant chemotherapy after pulmonary resection, while almost all patients (97%) received chemotherapy in the neoadjuvant group [28]. Similarly, the adjuvant setting sometimes requires dose reduction compared with the neoadjuvant setting; however, whether the full dose of chemotherapy is more effective when given with immunotherapeutic agents is unclear.

### 4.3. Necessity of Adjuvant Immunotherapy following Neoadjuvant Immunotherapy plus Surgical Resection

Whether adjuvant immunotherapy will improve patient outcomes after neoadjuvant immunotherapy plus surgical resection also remains unclear. In the CheckMate816 study (neoadjuvant nivolumab plus chemotherapy without adjuvant immunotherapy), patients who achieved a pathological complete response (pCR) showed excellent survival, suggesting that adjuvant immunotherapy may not be necessary for these patients. The potential role of adjuvant immunotherapy in patients who did not achieve pCR will be discussed in later sections.

## 5. Evidence of Neo-Adjuvant Immunotherapies

Neoadjuvant treatment with ICI includes ICI monotherapy, ICI plus ICI combination, ICI plus chemotherapy combination, and ICI plus chemoradiotherapy combination. Although neoadjuvant treatment using ICI monotherapy has shown some therapeutic efficacy, the most attention has been paid to the ICI plus chemotherapy strategies including perioperative (adjuvant ICI monotherapy in addition to neoadjuvant ICI plus chemotherapy) treatments.

One of the main purposes of neoadjuvant treatment is to reduce the tumor burden (primary tumor and/or metastatic lymph nodes), reduce the difficulty of the surgical procedure, avoid pneumonectomy, and/or improve the likelihood of complete resection. The other important purpose is the elimination of micrometastases by the earliest systemic treatment. In neoadjuvant studies, pCR and MPR data have been reported as some of the important parameters of efficacy. However, it should be noted that important elements for this calculation might be different between studies, e.g., pathological evaluation methods and formulas for calculation (whether the denominator of the equation is the total number of enrolled patients or the number of patients who underwent surgical resection).

### 5.1. ICI Monotherapies

The first piece of evidence regarding the usefulness of perioperative immunotherapies for NSCLC was reported by Forde PM, et al. in 2017 [5]. Nivolumab was administered only twice before surgery; however, it induced a MPR in 45% (9 of 20) of resected tumors. Responses occurred in both PD-L1-positive and -negative tumors, and a high tumor mutation burden (TMB) was associated with pathological response. The 5-year follow-up data of this study were recently reported and showed that eight of nine (89%) patients with MPR were alive and disease-free. Additionally, pre-treatment tumor PD-L1 positivity (TPS ≥ 1%) was associated with favorable recurrence-free survival (RFS) (HR, 0.36, 95% CI, 0.07–1.85) [29]. However, subsequent studies of neoadjuvant ICI monotherapies, using atezolizumab (with/without adjuvant atezolizumab [30]) or durvalumab [31], reported lower pCR and/or MPR results compared with the CheckMate 159 trial (Table 1). In these trials, surgical resection was performed in 88–93% of patients, and complete resection (R0) was achieved in 76–89% of cases. The pCR rates ranged from 0% to 10%. One study showed that the addition of radiotherapy to ICI monotherapy may improve the pathological response compared with ICI monotherapy alone (Table 1) [32].

### 5.2. ICI Combinations

In treatment of advanced-stage NSCLC, ICI combination therapies such as nivolumab plus the anti-CTLA-4 antibody ipilimumab showed superior efficacy over chemotherapy irrespective of the PD-L1 status (e.g., CheckMate 227) [35]. Therefore, it is reasonable to test the efficacy of such ICI combination therapies in neoadjuvant settings. However, one pilot trial of neoadjuvant nivolumab plus ipilimumab was terminated early by investigator consensus (after 9 of 15 patients were enrolled) because of toxicity [36]. On the other hand, the NEOSTAR randomized phase II trial successfully compared nivolumab alone with nivolumab plus ipilimumab in the neoadjuvant setting. Nivolumab and nivolumab plus ipilimumab treatments resulted in 22% (5/23) and 38% (8/21) MPR rates, respectively [37]. Additionally, nivolumab plus ipilimumab resulted in a higher pCR rate compared with nivolumab alone (29% vs. 9%, respectively, Table 2) and greater frequencies of effector, tissue-resident memory, and effector memory T cells. In a pilot analysis of microbiota, the abundance of *Ruminococcus* and *Akkermansia* spp. was associated with MPR in the nivolumab plus ipilimumab treatment group. The results of the nivolumab plus ipilimumab arm in the CheckMate 816 study (discussed later), although terminated early, may support the evaluation of the potential usefulness of this ICI combination regimen in the neoadjuvant setting.

There are many immune checkpoint molecules on the surface of immune cells in addition to PD-1 and CTLA-4 (Figure 2), and several novel immune checkpoint inhibitors are now being developed as anti-cancer agents [38]. Some of these novel immune checkpoint inhibitors have been tested in the neoadjuvant setting. For example, the NEOpredict-Lung trial, a phase II study, is comparing nivolumab monotherapy vs. nivolumab plus relatlimab, an anti-LAG-3 antibody [39]. The NeoCOAST trial is a phase II study evaluating durvalumab alone and in combination with the novel immunotherapeutic agents oleclumab, an anti-CD73 monoclonal antibody, monalizumab, an anti-NKG2A monoclonal antibody, and dambatilsen, an anti-STAT3 antisense oligonucleic acid [40]. While it was not possible to statistically compare these treatment groups, all combination therapies demonstrated a higher MPR compared with durvalumab monotherapy.

**Table 2:** Clinical trials of neoadjuvant ICI combinations.

Phase: Trial Name (Registry ID) Study Start Date (Reference)	Target Stage	N	ICI	pCR Rate	MPR Rate	DFS/EFS PFS/RFS	OS
pII: NEOSTAR (NCT03158129) June 2017 [37]	I–IIIA	44	N vs. N + I	N: 9% N + I: 29%	N: 22% N + I: 38%	RFS Median was not reached	Median was not reached
pII: NEOpredict-Lung (NCT04205552) March 2020 [39]	IB–IIIA	60	N vs. N + R	pCR or MPR rates N: 28% N + R: 32%		both arms 12 m DFS 91% [95% CI: 78–97%]	both arms 12 m OS 96% [95% CI: 83–99%]
pII: JHU and MSKCC Study (NCT02259621) September 2014 [36]	IB (≥4 cm)–IIIA	9	N + I (+two additional doses of N)	22%	NA	2y RFS 33%	NA

ICI: immune checkpoint inhibitor, MPR: major pathological response (tumors with no more than 10% viable tumor cells), pCR: pathological complete response, DFS: disease-free survival, EFS: event-free survival, PFS: progression-free survival, RFS: recurrence-free survival, OS: overall survival, NA: not available, I: ipilimumab, N: nivolumab, R: relatlimab.

### 5.3. Combination Therapies with ICI plus Cytotoxic Chemotherapies

Greater efficacies of PD-1 or PD-L1 inhibitors plus cytotoxic chemotherapies were reported in several phase I and II trials in the neoadjuvant setting [41,42,43,44], and these findings were further confirmed in the CheckMate816 trial [23]. Theoretically, immunotherapy will have additive effects because cytotoxic drugs will cause increased neoantigen release around the tumors by killing cancer cells. Furthermore, the addition of cytotoxic agents may prevent hyper-progressive disease [45], which may cause inoperability if it occurs in a neoadjuvant setting.

In the phase III Checkmate 816 trial, neoadjuvant nivolumab plus platinum-doublet (CDDP-based/CBDCA-based therapy) reduced the risk of relapse or death by 37% compared with chemotherapy alone (hazard ratio for event-free survival (EFS), 0.63; *p* = 0.005) [23]. The pCR and MPR rates were significantly better in the nivolumab-plus-chemotherapy group than in the chemotherapy group. In subgroup analyses, patients with cStage IIIA NSCLC (hazard ratio: 0.54, 95% CI 0.37–0.80) or those with high PD-L1 expression (TPS 50% or higher, hazard ratio: 0.24, 95% CI 0.10–0.61) showed better EFS with the addition of nivolumab, while the impact of the addition of nivolumab was not so high in the counterpart subgroups (patients with cStage IB–II NSCLC, hazard ratio: 0.87, 95% CI 0.48–1.56, and those with negative PD-L1 expression, hazard ratio: 0.85, 95% CI 0.54–1.32) (Table 3).

### 5.4. ICI Sandwich Therapies (Neoadjuvant ICI plus Chemotherapy Followed by Adjuvant ICI Monotherapy)

In many phase III trials using ICI plus cytotoxic chemotherapy as neoadjuvant treatment, postoperative administration of ICI monotherapy is also planned (often called perioperative treatments or sandwich regimens). In the NADIM single-arm phase II trial, the efficacy and safety of this strategy were reported. Additionally, several biomarker analyses using tumor specimens and/or blood samples were performed in this trial to identify patients who will benefit from neoadjuvant ICI treatment or patients who have poor prognosis after neoadjuvant ICI treatment (patients who may need adequate adjuvant treatment).

The initial results of several phase III studies of perioperative ICI treatments, including the AEGEAN trial [46], Neotoarch trial [47], KEYNOTE-671 trial [22], and NADIM II trial (a randomized phase II trial) [48], were recently reported (Table 4). In these trials, neoadjuvant ICI plus chemotherapy significantly improved the pCR and MPR rates and EFS (and OS in some trials) over neoadjuvant chemotherapy plus placebo. Similar to the results in the CheckMate816 trial, a higher proportion of patients achieved pCR after neoadjuvant ICI plus chemotherapy and these patients had excellent EFS. Therefore, an important clinical question remains as to whether adjuvant ICI monotherapy is necessary (or has some role) in patients who do not achieve pCR. In the subgroup analysis in some perioperative ICI trials, the survival curves for the ICI combination group were much better than those for the placebo group among patients who did not achieve pCR (at least compared with similar data from the CheckMate816 trial). Such data may suggest that there are some patients who would benefit from adjuvant ICI monotherapy after neoadjuvant ICI plus chemotherapy and surgical resection. This is an intriguing result because this suggests that a drug that fails to eradicate all cancer cells preoperatively with chemotherapy can eradicate cancer cells by monotherapy postoperatively. This biological paradox and the financial toxicity when adjuvant ICI monotherapy is applied to all patients should be explored in future studies.

### 5.5. Combination Therapies with Chemoradiotherapy (CRT)

Several studies have explored the addition of radiotherapy in ICI plus cytotoxic chemotherapies [51,52,53]. Compared with the neoadjuvant trials described above, these trials are mainly focused on patients with more advanced stages (for example, clinical N2 and/or stage III disease only) (Table 5). Radiotherapy is expected to increase the local control of mediastinal lymph node metastases; furthermore, it is expected that the abscopal effect of radiotherapy may promote anti-tumor immune reactions of ICI treatment [54].

There is currently insufficient data to support the use of ICI plus chemoradiotherapy over ICI plus chemotherapy because it is likely that treatment-related and/or surgery-related adverse events may increase after neoadjuvant ICI plus chemoradiotherapies. Further study is essential to examine if this combination therapy is useful to reduce the risk of inoperability when used for patients with marginally resectable tumors before treatment.

## 6. Evidence of Adjuvant Immunotherapies

Unlike neoadjuvant strategies for which pathologic response can be evaluable, the efficacy of adjuvant treatment can only be validated based on DFS data. Therefore, the form of large phase III trials is necessary to demonstrate its statistically significant benefit. To date, results from the IMpower010 and PEARLS trials have been published.

The efficacy of adjuvant ICI monotherapy, after adjuvant platinum-doublet chemotherapy, was reported by the IMpower010 study [3]. This study demonstrated the efficacy of adjuvant atezolizumab for 1 year compared with the best supportive care in patients with pathologic stage II–IIIA NSCLC with PD-L1 TPS ≥ 1% (HR for disease-free survival (DFS): 0.66 [95% CI: 0.50–0.88; *p* = 0.0039]). The highest improvement in OS was seen in stage II–IIIA patients with tumors that expressed PD-L1 TPS ≥ 50% (HR = 0.43, 95% CI: 0.24–0.78) [56]. An interim analysis of the Phase III PEARLS/KEYNOTE-091 trial evaluating pembrolizumab as adjuvant chemotherapy in patients with pathologic stage IB-IIIA NSCLC was also reported. Postoperative adjuvant pembrolizumab maintained a favorable improvement in DFS at 3 years. The median DFS for the overall population was 53.6 months in the pembrolizumab group and 42.0 months in the placebo group (hazard ratio [HR]: 0.76, 95% confidence interval [CI]: 0.63–0.91, *p* = 0.0014) [39]. However, the efficacy of adjuvant pembrolizumab was not associated with PD-L1 expression status, and the detailed mechanism of this phenomenon is unclear. There are several ongoing large clinical trials in the adjuvant setting such as ANVIL, BR31, and ALCHEMIST trials (Table 6).

## 7. Implications for Clinical Practice

These neoadjuvant, adjuvant, or perioperative clinical trials have demonstrated dramatic efficacy of ICI therapy over conventional standard treatment for some NSCLC patients. Since the drugs available at this time vary from country to country, we must take into account the results of these clinical trials and provide the best possible treatment for NSCLC patients using the available drug(s). In addition, these treatments have the potential, although not very likely, for unfavorable outcomes (e.g., risk of inoperability after neoadjuvant treatment or risk of developing severe irAE), so treatment strategies should be determined with patients considering their philosophy as well as levels of understanding of the treatment.

Our personal opinions, in situations where several treatment options are considerable, include neoadjuvant ICI treatment (especially ICI plus chemotherapy) would be superior to upfront surgery or CRT followed by surgery in patients with potential systemic disease (e.g., cN2 disease). On the other hand, neoadjuvant CRT, as a powerful tool for local control, remains a useful treatment option when surgical margins will be limited due to direct invasion of the primary tumor, such as tumors invading the chest wall near the vertebral body or superior sulcus tumors. In such cases, we consider that adjuvant atezolizumab would be one of the treatment options after surgical resection.

It should be noted that most of the currently available data have been obtained from patients participating in clinical trials. Therefore, there are insufficient data on the efficacy and safety of neoadjuvant, adjuvant, and perioperative treatment with ICIs in patients who do not meet the eligibility criteria for clinical trials (e.g., patients with autoimmune diseases) but who are considered at high risk of recurrence. Therefore, it is necessary to accumulate data on these patients as real-world data by conducting clinical practice with paying sufficient attention to safety.

## 8. Future Directions

Definitive evidence has shown the efficacy of neoadjuvant, adjuvant, and perioperative treatments using ICIs to improve patient survival and even exhibit curative effects in some patients. However, many challenges remain to be overcome. The most important and urgent need is to identify biomarkers to select appropriate patients who should receive these immunotherapies. Because of irAEs, some patients will require permanent therapy. Furthermore, some patients may experience lethal irAEs such as myocarditis. Therefore, ideally, immunotherapy must be applied to patients who have a high risk of recurrence and who will receive benefit from the therapy. Here we will summarize the attempts to develop such prognostic and predictive biomarkers.

### 8.1. Prognostic Biomarkers

The pathological stage, as well as the clinical stage in the case of neoadjuvant treatment, is one of the most important prognostic biomarkers for NSCLC. Therefore, neoadjuvant, adjuvant, and perioperative treatments are defined on the basis of these stages in several guidelines. However, the current TNM staging is determined on the basis of the spread of tumor cells but does not reflect the biology of tumor cells. Therefore, many groups have analyzed and reported the expression status of numerous genes, proteins, or clinicopathological markers as prognostic factors [58,59,60,61]. However, none have been used in clinical practice for the treatment of early-stage NSCLCs.

Recent evidence supports that circulating tumor DNA (ctDNA) detection after pulmonary resection indicates the presence of minimal residual disease (MRD), which is directly related to disease recurrence. Several retrospective studies have observed a high risk of recurrence in patients with positive ctDNA after pulmonary resection [62,63,64]. Some of these studies also reported that adjuvant cytotoxic chemotherapy may have some benefit in patients with positive ctDNA but not in patients without detectable ctDNA [63,64]. However, in the IMpower010 study of adjuvant atezolizumab compared with the best supportive care, both patient groups, those with and without ctDNA, benefited from adjuvant atezolizumab (although survival was better in patients without detectable ctDNA) [65]. Efforts are now being performed to increase the sensitivity of MRD detection to classify patients into two groups: Those with a high risk of recurrence who should receive adjuvant treatment and those who can be cured by surgery alone. However, a strategy using MRD detection is not applicable to deciding on neoadjuvant immunotherapy.

### 8.2. Predictive Biomarkers

There are several biomarkers to identify patients who may benefit from immunotherapies, including the PD-L1 status of tumor cells (and immune cells in the tumor microenvironment), tumor mutation burden, invasion of CD8-positive cells, and the absence of suppressive immune cells. Among these potential markers, the PD-L1 TPS was reported as a clinically meaningful biomarker to predict the efficacies of adjuvant, neoadjuvant, and/or perioperative anti-PD-1/PD-L1 antibodies, as reported in many trials including IMpower010, CheckMate816, and AEGEAN studies [3,23,24].

On the other hand, many studies did not support the usefulness of TMB as a predictive biomarker in perioperative settings. However, responses were also noted in patients with negative or low PD-L1 expression. Additionally, some patients with high PD-L1 expression also experience disease recurrence. Therefore, the identification of biomarkers that enhance the predictive impact of PD-L1 TPS is highly anticipated. Candidate biomarkers include tumor-infiltrating lymphocytes, immune scores, tertiary lymphoid structure, and gut microbiota.

## 9. Conclusions

The clinical application of ICIs is now dramatically changing the treatment of early-stage NSCLC patients who are candidates for surgical resection. Many neoadjuvant, adjuvant, and perioperative ICI treatments have shown significant survival benefits in these patients. In addition, ongoing clinical trials will provide more options for perioperative systemic treatments. As a future challenge, we have to collect real-world patients’ data to complement clinical trial data; in addition, prognostic and/or predictive biomarker studies are desirable for optimal individual treatment for resectable NSCLC patients. 

## Figures and Tables

**Table 1:** Clinical trials of neoadjuvant ICI monotherapies.

Phase: Trial Name (Registry ID) Study Start Date (Reference)	Target Stage	Inclusion Criteria (Biomarker)	N	ICI	Response Rate	pCR Rate	MPR Rate	DFS/EFS PFS/RFS	OS
pII: CheckMate 159 September 2014 (NCT02259621) [29]	I (>4 cm)–IIIA	-	21	N	NA	10%	45%	5 y RFS 60%	5 y OS 80%
pII: LCMC3 (NCT02927301) April 2017 [30]	IB–IIIB	w/o EGFR/ALK	181	A	NA	6% [95% CI: 3–11]	20% [95% CI: 14–28]	3 y DFS: 72% [95% CI: 62–79]	3 y OS: 80% [95% CI: 71–87]
pII: PRINCEPS (NCT02994576) December 2016 [33]	IA (≥2 cm)–IIIA	-	30	A	NA	0%	14%	NA	NA
pII: IONESCO (NCT03030131) January 2017 [31]	IB (≥4 cm)–IIIA (non N2)	-	46	D	PR: 9% SD: 78% PD: 13%	7%	19%	12 m DFS: 78.3%	12 m OS: 89.1%
pII: New York- Presbyterian and Weill Cornell Medical Center Study (NCT02904954) December 2016 [32]	I–IIIA	-	60	D vs. D + SBRT (8Gy × 3fr)	D PR: 3% SD: 80% PD: 10% D + SBRT PR: 50% SD: 50%	D: 0% D + SBRT: 27%	D: 6% D + SBRT: 26%	NA	NA
pII: POTENTIAL (jRCT2061180016) January 2019 [34]	I	*w*/*o* EGFR or ALK or ROS1	50	N	Ongoing	Ongoing	Ongoing	Ongoing	Ongoing

ICI: immune checkpoint inhibitor, pCR: pathological complete response, MPR: major pathological response (tumors with no more than 10% viable tumor cells), DFS: disease-free survival, EFS: event-free survival, PFS: progression-free survival, RFS: recurrence-free survival, OS: overall survival, NA: not available, SBRT: stereotactic body radiotherapy, A: atezolizumab, D: durvalumab, N: nivolumab, P: pembrolizumab.

**Table 3:** Clinical trials of neoadjuvant ICI plus chemotherapy.

Phase: Trial Name (Registry ID) Study Start Date (Reference)	Target Stage	N	ICI	Response Rate	pCR Rate	MPR Rate	DFS/EFS PFS/RFS	OS
pII: Sidney Kimmel Study (NCT03366766) December 2017 [42]	IB (≥4 cm)–IIIA	13	N + CDDP + PEM or GEM	CR: 8% PR: 38% SD: 54%	38%	85%	NA	NA
pII: Columbia University Study (NCT02716038) June 2016 [41]	IB–IIIA	30	A + CBDCA + nab-PTX	PR: 63% SD: 30% PD: 7%	33%	57%	17·9 m [95% CI: 14.3–not reached]	Median not reached
pIII: CheckMate 816 (NCT02998528) March 2017 [23]	IB (>4 cm)–IIIA	358	N + PT-DC vs. PT-DC	NA	N + PT-DC: 24% PT-DC: 2.2%	N + PT-DC: 36.9% PT-DC: 8.9%	Median EFS N + PT-DC: 31.6 m [95% CI: 30.2– not reached] PT-DC: 20.8 m [95% CI: 14.0–26.7] HR: 0.63 [97.38% CI: 0.43–0.91] *p* = 0.005	Median not reached

ICI: immune checkpoint inhibitor, MPR: major pathological response (tumors with no more than 10% viable tumor cells), pCR: pathological complete response, DFS: disease-free survival, EFS: event-free survival, PFS: progression-free survival, RFS: recurrence-free survival, OS: overall survival, NA: not available, A: atezolizumab, D: durvalumab, N: nivolumab, CBDCA: carboplatin, CDDP: cisplatin, GEM: gemcitabine, nab-PTX: nab-paclitaxel, PEM: pemetrexed, PT-DC: platinum-based doublet chemotherapy.

**Table 4:** Clinical trials on ICI sandwich treatments.

Phase: Trial Name (Registry ID) Study Start Date (Reference)	Target Stage	N	ICI	Response Rate	pCR Rate	MPR Rate	DFS/EFS PFS/RFS	OS
pIII: AEGEAN (NCT03800134) December 2018 [24]	IIA–IIIB	740	<Neoadjuvant> D or Placebo + PT-DC ↓ <Adjuvant> D or Placebo	NA	D + Chemo: 17.2% Chemo: 4.3% (*p* = 0.000036)	NA	Median EFS D + Chemo: NR [95% CI: 31.9–NR] Chemo: 25.9 m [95% CI: 18.9–NR] HR: 0.68 [95% CI: 0.53–0.88], *p* = 0.003902	NA
pIII: Neotorch trial (NCT04158440) April 2020 [47]	II–III	404	<Neoadjuvant> T or Placebo + PT-DC ↓ <Adjuvant> T or Placebo + PT-DC either single agent T or placebo	NA	T: 24.8% Placebo: 1.0%	T: 48.5% Placebo: 8.4%	EFS HR = 0.40 [95% CI: 0.277–0.565, *p* < 0.0001]	NA
pIII: KEYNOTE-671 (NCT03425643) April 2018 [22]	II, IIIA or IIIB (N2)	797	<Neoadjuvant> P or PT-DC ↓ <Adjuvant> P or Placebo	NA	P: 18.1% placebo: 4.0%	P: 30.2% placebo: 11.0%	24 m EFS P: 62.4% placebo: 40.6% HR = 0.58 [95% CI: 0.46–0.72, *p* < 0.001]	24 m OS P: 80.9% placebo: 77.6% HR = 0.58 [*p* = 0.02, which did not meet the significance criterion]
pII: NADIM (NCT03081689) April 2017 [44]	IIIA	46	<Neoadjuvant> N + PTX + CBDCA ↓ <Adjuvant> N	CR: 4% PR: 72% SD: 24%	63%	83%	PFS 12 m: 95.7% [95% CI: 83.7–98.9] 18 m: 87.0% [95% CI: 73.3–93.9] 24 m: 77.1% [95% CI: 59.9–87.7]	12 m: 97.8% [95% CI: 85.5–98.7] 18 m: 93.5% [95% CI: 81.1–97.8] 24 m: 89.9% [95% CI: 74.5–86.2]
pII: NADIM II (NCT03838159) May 2019 [48]	Resectable IIIA or IIIB	86	<Neoadjuvant> N +PTX + CBDCA or PTX + CBDCA ↓ <Adjuvant> N or follow up	N + Chemo: 75% Chemo: 48% RR: 1.56 [95% CI: 1.04–2.34]	N + Chemo: 37% Chemo: 7% RR: 5.34 [95% CI: 1.34–21.23] *p* = 0.02	N + Chemo: 53% Chemo: 14% RR: 3.82 [95% CI: 1.49–9.79]	24 m PFS N + Chemo: 67.2% Chemo: 40.9% HR = 0.47 [95% CI: 0.25–0.88]	24 m OS N + Chemo: 85% Chemo: 63.6% HR = 0.43 [95% CI: 0.19–0.98]
pII: SAKK 16/14 (NCT02572843) June 2016 [43]	IIIA (N2)	67	<Neoadjuvant> CDDP + DTX→ D ↓ <Adjuvant> D	CR: 7% PR: 52% SD: 26% PD: 7%	18%	62%	12 m EFS 73% [two-sided 90%CI: 63–82]	Median not reached
pIII: IMpower030 (NCT03456063) April 2018 [49]	II, IIIA, or select IIIB (T3N2)	302	<Neoadjuvant> A or Placebo+ PT-DC ↓ <Adjuvant> A or BSC/scheduled observational follow-up	Ongoing	Ongoing	Ongoing	Ongoing	Ongoing
pIII: CheckMate-77T (NCT04025879) November 2019 [50]	IIA–IIIB (T3N2 only)	452	<Neoadjuvant> N + CBDCA or PT-DC ↓ <Adjuvant> N	Ongoing	Ongoing	Ongoing	Ongoing	Ongoing

ICI: immune checkpoint inhibitor, MPR: major pathological response (tumors with no more than 10% viable tumor cells), pCR: pathological complete response, DFS: disease-free survival, EFS: event-free survival, PFS: progression-free survival, RFS: recurrence-free survival, OS: overall survival, NA: not available, RR: relative risk, D: durvalumab, N: nivolumab, P: pembrolizumab, T: toripalimab, CBDCA: carboplatin, DTX: docetaxel, PT-DC: platinum-based doublet chemotherapy, PTX: paclitaxel. The arrow indicates the order of the treatment.

**Table 5:** Clinical trials on combination therapies with ICI plus chemoradiotherapy.

Phase: Trial Name (Registry ID) Study Start Date (Reference)	Target Stage	N	ICI	Response Rate	pCR Rate	MPR Rate	DFS/EFS PFS/RFS	OS
pII: SQUAT trial (WJOG12119L) December 2019 [51]	IIA–IIIB (N2)	30	<Neoadjuvant> D + wPTX + CBDCA + Radiotherapy (50Gy2G × 25fr) ↓ <Adjuvant> D	PR: 47% SD: 47% PD: 14% PD: 6%	23% [95% CI: 9–42]	63% [95% CI: 44–80]	NA	NA
pI: Cleveland Clinic Study (NCT02987998) May 2017 [53]	IIIA	9	<Neoadjuvant> P + CDDP + VP-16 + Radiotherapy (45Gy × 25fr) ↓ <Adjuvant> P	PR: 75% PD: 25%	67%	NA	6 m PFS 55.6% [95% CI: 31–99]	3y OS 64%: [95% CI: 39–100]
pII: The INCREASE trial (EudraCT-Number: 2019–003454-83) December 2019 [55]	T3-4 (N0–2)	26	<Neoadjuvant> [Day1] I + N + PT-DC + Radiotherapy * [Day22] N + PT-DC + Radiotherapy *	PR: 12.5% SD: 87.5%	63%	79%	NA	NA
pIb: Yonsei University Study (NCT03694236) February 2019 [52]	III	30	<Neoadjuvant> D + wPTX + CBDCA + Radiotherapy (45Gy × 25fr) ↓ <Adjuvant> D	ORR: 50%	41%	74%	NA	NA
pII: SAKK 16/18 (NCT04245514) July 2020	IIA–IIIB (N2)	90	<Neoadjuvant> D + wPTX + CBDCA + Radiotherapy (2Gy × 20fr or 5Gy × 5fr or 8Gy × 3fr) ↓ <Adjuvant> D	Ongoing	Ongoing	Ongoing	Ongoing	Ongoing

ICI: immune checkpoint inhibitor, MPR: major pathological response (tumors with no more than 10% viable tumor cells), pCR: pathological complete response, ORR: objective response rare, DFS: disease-free survival, EFS: event-free survival, PFS: progression-free survival, RFS: recurrence-free survival, OS: overall survival, NA: not available, D: durvalumab, P: pembrolizumab, I: ipilimumab, N: nivolumab, CBDCA: carboplatin, CDDP: cisplatin, wPTX: weekly paclitaxel, VP-16: etoposide. *: once daily dose of 2Gy. The arrow indicates the order of the treatment.

**Table 6:** Clinical trials on adjuvant ICI.

Phase: Trial Name Study Start Date (Registry ID) (Reference)	Target Stage	N	ICI	DFS/EFS PFS/RFS	OS
pIII: IMpower010 (NCT02486718) October 2015 [3,57]	IB (≥4 cm)–IIIA	1005	A vs. BSC	DFS in stage II–IIIA & PD-L1 TPS ≥ 1% HR = 0.66 [95% CI: 0.50–0.88, *p* = 0.0039] DFS in stage II–IIIA & PD-L1 TPS ≥ 50% HR = 0.47 [95% CI: 0.29–0.75]	OS in stage II–IIIA & PD-L1 TPS ≥ 1% HR = 0.71 [95% CI: 0.49–1.03, *p* = 0.067] OS in stage II–IIIA & PD-L1 TPS ≥ 50% HR = 0.43 [95% CI: 0.24–0.78, *p* = 0.0045]
pIII: PEARLS/ KEYNOTE-091 (NCT02504372) November 2015 [4]	IB (≥4 cm) –IIIA	1177	P vs. Placebo	median DFS HR 0.76; [95% CI 0.63–0.91, *p* = 0.0014]	3 y OS HR 0.87; [95% CI 0.67–1.15, *p* = 0.17]
pIII: ANVIL (NCT02595944) July 2016	IB (≥4 cm)–IIIA	903	N vs. Placebo	Ongoing	Ongoing
pIII: BR31 (NCT02273375) February 2015	IB (≥4 cm) –IIIA	1360	D vs. Placebo	Ongoing	Ongoing
pIII: ALCHEMIST-IO (NCT04267848) June 2020 [57]	IB (T≥4 cm)– IIIA	1263	<Arm A> PT-DC ↓ ± PORT ↓ Observation <Arm B> PT-DC ↓ ± PORT ↓ P <Arm C> PT-DC + P ↓ ± PORT ↓ P	Ongoing	Ongoing

ICI: immune checkpoint inhibitor, MPR: major pathological response (tumors with no more than 10% viable tumor cells), pCR: pathological complete response, DFS: disease-free survival, EFS: event-free survival, PFS: progression-free survival, RFS: recurrence-free survival, OS: overall survival, NA: not available, D: durvalumab, P: pembrolizumab, N: nivolumab, PT-DC: platinum-based doublet chemotherapy, BSC: best supportive care, PORT: postoperative radiation therapy. The arrow indicates the order of the treatment.

## Data Availability

All essential information will be available from the corresponding author upon reasonable request.
